# Association between dry eye and periodontal disease in community-dwelling Japanese adults: data from the Uonuma cohort study

**DOI:** 10.1186/s12903-023-03773-7

**Published:** 2024-01-08

**Authors:** Kaung Myat Thwin, Noboru Kaneko, Hikaru Okubo, Takayuki Yamaga, Kana Suwama, Akihiro Yoshihara, Masanori Iwasaki, Yumi Ito, Junta Tanaka, Ichiei Narita, Hiroshi Ogawa

**Affiliations:** 1 Division of Preventive Dentistry, Faculty of Dentistry & Graduate School of Medical and Dental Sciences, Niigata University, 2-5274, Gakkocho-dori, Chuo-ku, Niigata, 951-8514 Japan; 2 Department of Oral Health, School of Dentistry, Matsumoto Dental University, Shiojiri, Japan; 3 Division of Oral Science for Health Promotion, Faculty of Dentistry & Graduate School of Medical and Dental Sciences, Niigata University, Niigata, Japan; 4 Division of Preventive Dentistry, Department of Oral Health Science, Graduate School of Dental Medicine, Hokkaido University, Sapporo, Japan; 5 Department of Health Promotion Medicine, Graduate School of Medical and Dental Sciences, Niigata University, Niigata, Japan

**Keywords:** Dry eye, Periodontal disease, Adults, Uonuma cohort study, Japan

## Abstract

**Background:**

While research has explored the risk of periodontal disease in various eye conditions, the link between dry eye and periodontal disease remains underexplored, especially in Japanese adults. This study aims to investigate the association between dry eye and periodontal disease in community-dwelling Japanese adults.

**Methods:**

This study is a subset of the Uonuma cohort study, which includes Japanese adults aged 40 years and older residing in the Uonuma area of Niigata Prefecture, Japan. Participants completed a self-administered, paper-based questionnaire. Statistical analyses, including the chi-square test, independent t test, ANOVA test, and logistic regressions, were employed to assess the association of periodontal disease with independent variables.

**Results:**

Among 36,488 participants (average age 63.3 years, 47.4% men), 39.3% had a history of periodontal disease, and gender differences were statistically significant (p < 0.001). Significant associations were found between periodontal disease and dry eye diagnosis or symptoms. Univariable logistic regression revealed links between periodontal disease and age, gender, living status, alcohol consumption, remaining teeth, bite molar availability, and history of dry eye disease or symptoms. Multiple-adjusted regression found that doctor-diagnosed dry eye was associated with a higher likelihood of periodontal disease (odds ratio, 1.12; 95% confidence interval, 1.03–1.22). Participants who never experienced dryness or foreign body sensation had lower ORs of periodontal disease than those who always experienced such symptoms across all models.

**Conclusion:**

A significant correlation was found between dry eye and periodontal disease in Japanese adults. Regular check-ups, early detection, and effective management of both conditions are strongly recommended.

**Supplementary Information:**

The online version contains supplementary material available at 10.1186/s12903-023-03773-7.

## Background

Periodontal disease (PD) is a common disease experienced by an estimated 20.0–50.0% of the global population [1]. It can lead to the destruction of supporting tissues of the teeth in susceptible patients, potentially resulting in eventual tooth loss [2]. Additionally, it stands as a leading cause of tooth loss among Japanese adults affecting 32.4% of those aged 25–34 years, 49.5% of those aged 45–54 years, and 57.5% of those aged 65–74 years [3].

PD is associated with multiple systemic diseases including rheumatoid arthritis, diabetes, adverse pregnancy outcomes, and cardiovascular diseases [4, 5]. The long-term systemic inflammation caused by oral bacteria in patients with PD can affect the entire body through both infectious and inflammatory responses, potentially increasing the risk of systemic diseases [2, 6]. As a result, the impact of PD on general health has been increasing to focus on research interests. Acknowledging that PD is both preventable and irreversible, many countries have initiated efforts to reduce the burden of PD [7].

Similar to PD, several eye diseases are associated with systemic inflammatory pathways, and reports have linked them to PD [8, 9]. Among these diseases, dry eye disease (DED) is one of the most common ocular diseases worldwide, with an estimated prevalence of 11.6% [10]. This condition poses a growing public health concern due to its impact on visual function and quality of life [11]. Common causes of DED include aging, certain medical conditions such as Sjögren’s syndrome, and wearing contact lenses [10].

Sjögren’s syndrome is an autoimmune disease characterized by the cardinal symptoms of dry eye and dry mouth. Several studies have investigated the risk of PD in patients with Sjögren’s syndrome and those with dry mouth, indicating the common inflammatory pathways through systemic inflammatory responses and alterations in pro-inflammatory cytokines [2, 6, 9]. Similarly, DED is also characterized by elevated levels of inflammatory cytokines [10], hinting a potential systemic link between DED and PD. However, the available evidence concerning the association between these conditions remains limited. Furthermore, to the best of our knowledge, there is a lack of information regarding the relationship between DED and PD among Japanese adults. Therefore, this study aims to investigate the association between DED and PD in a community-dwelling Japanese adult population. We hypothesize that individuals with a history of dry eye disease or related symptoms will exhibit a higher prevalence of PD than those without any history of dry eye disease or symptoms.

## Methods

### Study design and participants

This study is a subset of the Uonuma cohort study, which targeted Japanese adults aged 40 years and older in the Uonuma area of Niigata Prefecture, Japan [12]. The study utilized the results of a self-administered questionnaire survey conducted between 2012 and 2014 as its baseline. All Japanese residents of Minami-Uonuma City and Uonuma City aged 40 years and older (n = 61,762) were invited to complete a questionnaire. Out of them, 39,774 residents (64.4%) responded to the questionnaire. After excluding individuals who withdrew their consent, or provided invalid responses, the study analyzed data from 36,488 residents (59.1%). This study protocol was approved by the Ethics Committee of Niigata University, Japan (Approval number. 2017-0071). All methods were carried out in accordance with the guidelines and regulations established in the Declaration of Helsinki. Participants who agreed to participate in the study signed their written consent forms.

### Data collection

Self-administered questionnaires enclosed in sealed envelopes with return envelopes were mailed to the residents in cooperation with the local government authorities. Information regarding their age and gender was obtained from the local government records. Participants completed and returned the questionnaires. The questionnaire included inquiries about body weight and height to calculate the body mass index, BMI (1: healthy, 2: underweight, 3: overweight, or 4: obese), living status (1: with 5 persons and above, 2: with 3–4 persons, 3: with 1–2 persons, or 4: alone), smoking habit (1: never, 2: former smoker, or 3: current smoker), drinking habit (1: never, 2: former drinker, or 3: current drinker), history of PD diagnosed by a dentist (1: no or 2: yes), number of remaining teeth (1: ≥20 teeth, 2: 10–19 teeth, 3: 1–9 teeth, 4: no natural teeth, or 5: don’t know), biting availability on right and left molars or with dentures (1: both sides, 2: one side, or 3: no side), history of DED diagnosed by a doctor (1: no or 2: yes), feeling dryness of eyes (1: never, 2: sometimes, or 3: always), and feeling a foreign body sensation in eyes (1: never, 2: sometimes, or 3: always).

### Statistical analysis

We performed statistical analyses using IBM SPSS Statistics for Windows, version 22.0 (SPSS; Chicago, IL, USA). Demographics and general data were reported using descriptive statistics. Participants were classified into two groups: those with a history of PD and those without such a history (0: no history of PD, 1: history of PD). The associations between periodontal disease and independent variables were examined using the chi-square test, independent t test, and ANOVA test. We also employed univariable and multivariable logistic regression analyses to assess the potential risk factors related to PD. Multivariable logistic regression was conducted across a series of models. Model 1 adjusted for sociodemographic variables such as age, gender, BMI, and living status. Model 2 further adjusted for behavioral factors, including smoking and drinking habits. Model 3 extended the adjustments to oral conditions such as the number of remaining teeth and the bite availability on the right and left molars or with dentures. Subsequently, we calculated the 95% confidence interval (95% CI) for each odds ratio (OR) in our logistic regression models. The threshold for statistical significance for all tests was set at p < 0.05.

## Results

The sample consisted of 36,488 participants, with an average age of 63.3 years, and 47.4% of them were men (n = 17,302). The mean number of present teeth (standard deviation) was 20.6 (9.3) in men and 21.0 (9.0) in women. The general characteristics of the participants are presented in Table 1. Overall, 39.3% of the participants had a history of PD diagnosed by a dentist, and there was a statistically significant difference in PD history between genders (p < 0.001). Gender exhibited a significant association with most variables in our study, excluding questions related to the history of dry eye disease or symptoms and smoking habits.

**Table 1:** General characteristics of the participants

Variables	Total n (%)	Gender, n (%)		*p* value
		Male (n = 17,302)	Female (n = 19,186)	
Age (years)				
40–49 50–59 60–69 70–79 ≥ 80	6035 (16.5) 8363 (22.9) 10,500 (28.8) 7114 (19.5) 4476 (12.3)	2862 (16.5) 4004 (23.1) 5286 (30.6) 3339 (19.3) 1811 (10.5)	3173 (16.5) 4359 (22.7) 5214 (27.2) 3775 (19.7) 2665 (13.9)	**< 0.001**
BMI^a^				
Healthy Underweight Overweight Obese	26,006 (71.3) 2767 (7.6) 6707 (18.4) 1008 (2.8)	12,416 (71.8) 871 (5.0) 3628 (21.0) 387 (2.2)	13,590 (70.8) 1896 (9.9) 3079 (16.0) 621 (3.2)	**< 0.001**
Living status				
With 5 persons or more With 3–4 persons With 1–2 persons Alone	9974 (27.3) 11,509 (31.5) 14,441 (39.6) 564 (1.5)	4799 (27.7) 5363 (31.0) 6842 (39.5) 298 (1.7)	5175 (27.0) 6146 (32.0) 7599 (39.6) 266 (1.4)	**0.008**
Smoking habit				
Never Former smoker Current smoker	18,733 (51.3) 10,683 (29.3) 7072 (19.4)	8921 (51.5) 5055 (29.2) 3335 (19.3)	9821 (51.2) 5628 (29.3) 3737 (19.5)	0.812
Drinking habit				
Never Former drinker Current drinker	12,592 (34.5) 1845 (5.1) 22,051 (60.4)	5988 (34.6) 930 (5.4) 10,384 (60.0)	6604 (34.4) 915 (4.8) 11,667 (60.8)	**0.022**
History of periodontal disease				
No Yes	22,143 (60.7) 14,345 (39.3)	10,024 (57.9) 7278 (42.1)	12,119 (63.2) 7067 (36.8)	**< 0.001**
Number of remaining teeth				
≥ 20 teeth 10–19 teeth 1–9 teeth No natural teeth Don’t know	21,925 (60.1) 5306 (14.5) 3356 (9.2) 1583 (4.3) 4318 (11.8)	10,071 (58.2) 2627 (15.2) 1604 (9.3) 761 (4.4) 2239 (12.9)	11,854 (61.8) 2679 (14.0) 1752 (9.1) 822 (4.3) 2079 (10.8)	**< 0.001**
Bite availability on molars				
Both sides One side No side	28,859 (79.1) 5510 (15.1) 2119 (5.8)	13,531 (78.2) 2585 (14.9) 1186 (6.9)	15,328 (79.9) 2925 (15.2) 933 (4.9)	**< 0.001**
History of dry eye diagnosis				
No Yes	33,971 (93.1) 2517 (6.9)	16,139 (93.3) 1163 (6.7)	17,832 (92.9) 1354 (7.1)	0.207
Feels dryness of eyes				
Never Sometimes Always	24,845 (68.1) 10,329 (28.3) 1314 (3.6)	11,780 (68.1) 4921 (28.4) 601 (3.5)	13,065 (68.1) 5408 (28.2) 713 (3.7)	0.428
Feels a foreign body in the eyes				
Never Sometimes Always	26,375 (72.3) 9508 (26.1) 605 (1.7)	12,514 (72.3) 4519 (26.1) 269 (1.6)	13,861 (72.2) 4989 (26.0) 336 (1.8)	0.337

BMI: body mass index (kg/m^2^); n: number

^a^BMI was classified as healthy: 18.5–24.9; underweight: <18.5; overweight: 25.0–29.9; obese: ≥30.0

We observed significant associations between individuals with and without PD and the diagnosis or symptoms of dry eye (Table 2). Among male participants, those who reported experiencing dryness (*p* = 0.041) or a foreign body sensation in their eyes (*p* = 0.034) exhibited significantly higher rates of PD history than those without such symptoms. However, these symptoms did not correlate with a history of PD in female participants. Conversely, among female participants, a diagnosis of DED by a doctor was significantly linked to a history of PD (*p* = 0.014).

**Table 2:** Association between history of periodontal disease and dry eye diagnosis or symptoms by gender

Variables	Total participants, n (%)		*p* value	Male participants, n (%)		*p* value	Female participants, n (%)		*p* value
	With PD (n = 14,345)	Without PD (n = 22,143)		With PD (n = 7278)	Without PD (n = 10,024)		With PD (n = 7067)	Without PD (n = 12,119)	
History of dry eye diagnosis									
No Yes	13,289 (92.6) 1056 (7.4)	20,682 (93.4) 1461 (6.6)	**0.005**	6763 (92.9) 515 (7.1)	9376 (93.5) 648 (6.5)	0.113	6526 (92.3) 541 (7.7)	11,306 (93.3) 813 (6.7)	**0.014**
Dry eye symptom: Feels dryness in eyes									
Never Sometimes Always	9666 (67.4) 4137 (28.8) 542 (3.8)	15,179 (68.5) 6192 (28.0) 772 (3.5)	**0.046**	4888 (67.2) 2116 (29.0) 274 (3.8)	6892 (68.8) 2805 (28.0) 327 (3.3)	**0.041**	4778 (67.6) 2021 (28.6) 268 (3.8)	8287 (68.4) 3387 (27.9) 445 (3.7)	0.540
Dry eye symptom: Feels a foreign body in the eyes									
Never Sometimes Always	10,361 (72.2) 3717 (25.9) 272 (1.9)	16,014 (72.3) 5796 (26.2) 333 (1.5)	**0.015**	5252 (72.2) 1892 (26.0) 134 (1.8)	7262 (72.4) 2627 (26.2) 135 (1.3)	**0.034**	5109 (72.3) 1820 (25.8) 138 (2.0)	8752 (72.2) 3169 (26.1) 198 (1.6)	0.239

PD: periodontal disease

Table 3 presents the associations between a history of PD and the diagnosis or symptoms of dry eye, stratified by age groups. In the participants aged 50–59 years, individuals who reported experiencing dryness in their eyes demonstrated significantly increased rates of PD history compared to those without those symptom(*p* = 0.004). Furthermore, in older adults aged 70–79 years, the occurrence of a foreign body sensation in their eyes exhibited a significant association with a history of PD (*p* = 0.004).

**Table 3:** Association between history of periodontal disease and dry eye diagnosis or symptoms by age

Variables	40–49 years, n (%)		50–59 years, n (%)		60–69 years, n (%)		70–79 years, n (%)		≥ 80 years, n (%)	
	With PD (n = 1738)	Without PD (n = 4297)	With PD (n = 3324)	Without PD (n = 5039)	With PD (n = 4798)	Without PD (n = 5702)	With PD (n = 3039)	Without PD (n = 4075)	With PD (n = 1446)	Without PD (n = 3030)
History of dry eye diagnosis										
No	1603 (92.2)	4008 (93.3)	3074 (92.5)	4714 (93.6)	4426 (92.2)	5313 (93.2)	2840 (93.5)	3817 (93.7)	1346 (93.1)	2830 (93.4)
Yes	135 (7.8)	289 (6.7)	250 (7.5)	325 (6.4)	372 (7.8)	389 (6.8)	199 (6.5)	258 (6.3)	100 (6.9)	200 (6.6)
*p* value	0.152		0.058		0.067		0.712		0.694	
Dry eye symptom: Feels dryness in eyes										
Never	1173 (67.5)	2940 (68.4)	2195 (66.0)	3497 (69.4)	3228 (67.3)	3904 (68.5)	2072 (68.2)	2745 (67.4)	998 (69.0)	2093 (69.1)
Sometimes	487 (28.0)	1207 (28.1)	993 (29.9)	1373 (27.2)	1400 (29.2)	1584 (27.8)	859 (28.3)	1196 (29.3)	398 (27.5)	832 (27.5)
Always	78 (4.5)	150 (3.5)	136 (4.1)	169 (3.4)	170 (3.5)	214 (3.8)	108 (3.6)	134 (3.3)	50 (3.5)	105 (3.5)
*p* value	0.181		**0.004**		0.266		0.538		0.999	
Dry eye symptom: Feels a foreign body in the eyes										
Never	1251 (72.0)	3103 (72.2)	2361 (71.0)	3641 (72.3)	3441 (71.7)	4116 (72.2)	2247 (73.9)	2909 (71.4)	1061 (73.4)	2245 (74.1)
Sometimes	452 (26.0)	1124 (26.2)	893 (26.9)	1322 (26.2)	1263 (26.3)	1493 (26.2)	739 (24.3)	1116 (27.4)	365 (25.2)	741 (24.5)
Always	35 (2.0)	70 (1.6)	70 (2.1)	76 (1.5)	94 (2.0)	93 (1.6)	53 (1.7)	50 (1.2)	20 (1.4)	44 (1.5)
*p* value	0.585		0.090		0.433		**0.004**		0.842	

PD: periodontal disease

The findings presented in Table 4 represents the results of the univariable logistic regression analysis for PD. Significant associations with PD were found for sociodemographic factors such as age, gender, and living status. Smoking habits did not influence the likelihood of PD in our study, but alcohol consumption was significantly associated with PD. Participants with ≥ 20 remaining teeth were less likely to have PD than those with 10–19 teeth (OR 1.78; 95% CI, 1.67–1.89), 1–9 teeth (OR 1.83; 95% CI, 1.70–1.97), and no natural teeth (OR 1.42; 95% CI, 1.28–1.58). Additionally, molar bite availability emerged as a significant factor associated with PD history. A history of DED diagnosed by a doctor was associated with a higher likelihood of PD (OR 1.13; 95% CI, 1.04–1.22). Similarly, dry eye symptoms, including dryness and a foreign body sensation, were significantly linked to PD.

**Table 4:** Univariable logistic regression analysis for periodontal disease

Variables	Number of Individuals with PD (%)	Crude OR (95% CI)	*p* value
Age (years)			
40–49	1738 (28.8)	*Ref*	
50–59 60–69 70–79 ≥ 80	3324 (39.7) 4798 (45.7) 3039 (42.7) 1446 (32.3)	1.63 (1.52–1.75) 2.08 (1.94–2.23) 1.84 (1.71–1.98) 1.18 (1.09–1.28)	**< 0.001** **< 0.001** **< 0.001** **< 0.001**
Gender			
Male	7278 (42.1)	*Ref*	
Female	7067 (36.8)	0.80 (0.77–0.84)	**< 0.001**
BMI			
Healthy	10,218 (39.3)	*Ref*	
Underweight Overweight Obese	1054 (38.1) 2684 (40.0) 389 (38.6)	0.95 (0.88–1.03) 1.03 (0.98–1.09) 0.97 (0.85–1.11)	0.219 0.277 0.655
Living status			
With 5 persons or more	3776 (37.9)	*Ref*	
With 3–4 persons With 1–2 persons Alone	4371 (38.0) 5951 (41.2) 247 (43.8)	1.01 (0.95–1.06) 1.15 (1.09–1.21) 1.28 (1.08–1.52)	0.856 **< 0.001** **0.005**
Smoking habit			
Never	7408 (39.5)	*Ref*	
Former smoker Current smoker	4146 (38.8) 2791 (39.5)	0.99 (0.94–1.05) 0.97 (0.92–1.02)	0.907 0.214
Drinking habit			
Never	4839 (38.4)	*Ref*	
Former drinker Current drinker	777 (42.1) 8729 (39.6)	1.05 (1.00–1.10) 1.17 (1.06–1.29)	**0.034** **0.002**
Number of remaining teeth			
≥ 20 teeth	7848 (35.8)	*Ref*	
10–19 teeth 1–9 teeth No natural teeth Don’t know	2640 (49.8) 1695 (50.5) 700 (44.2) 1462 (33.9)	1.78 (1.67–1.89) 1.83 (1.70–1.97) 1.42 (1.28–1.58) 0.92 (0.86–1.28)	**< 0.001** **< 0.001** **< 0.001** 0.152
Bite availability on molars			
Both sides	10,660 (36.9)	*Ref*	
One side No side	2616 (47.5) 1069 (50.4)	1.54 (1.46–1.64) 1.74 (1.59–1.89)	**< 0.001** **< 0.001**
History of dry eye diagnosis			
No	13,289 (39.1)	*Ref*	
Yes	1056 (42.0)	1.13 (1.04–1.22)	**0.005**
Feels dryness of eyes			
Never	9666 (38.9)	*Ref*	
Sometimes Always	4137 (40.1) 542 (41.2)	1.10 (0.99–1.23) 1.05 (1.00–1.10)	0.090 **0.045**
Feels a foreign body in the eyes			
Never	10,361 (39.3)	*Ref*	
Sometimes Always	3712 (39.0) 272 (45.0)	0.99 (0.94–1.04) 1.26 (1.07–1.49)	0.678 **0.005**

BMI: body mass index (kg/m^2^); CI: confidence interval; OR: odds ratio; PD: periodontal disease; Ref: reference

^a^BMI was classified as healthy: 18.5–24.9; underweight: <18.5; overweight: 25.0–29.9; obese: ≥30.0

Table 5 shows the adjusted ORs for PD. In the variable “history of DED diagnosed by a doctor”, the multiple adjusted ORs for PD were 1.13 (95% CI, 1.04–1.23) for model 1, 1.13 (95% CI, 1.03–1.23) for model 2, and 1.12 (95% CI, 1.03–1.22) for model 3. Furthermore, participants who reported never experiencing dryness or a foreign body sensation in their eyes were significantly less likely to have PD than those who always experienced these symptoms in all models. Conversely, among male participants, only the variable “feeling a foreign body sensation in their eyes” was significantly associated with PD in all models (Supplementary Table 1). Female participants with a history of DED diagnosis were more likely to have PD than those without a DED diagnosis in all models (Supplementary Table 2).

**Table 5:** Multivariable logistic regression analysis for periodontal disease

Variables	Model 1^a^	*p* value	Model 2^b^	*p* value	Model 3^c^	*p* value
	Adjusted OR (95%CI)		Adjusted OR (95%CI)		Adjusted OR (95%CI)	
History of dry eye diagnosis						
No	*Ref*		*Ref*		*Ref*	
Yes	1.13 (1.04–1.23)	**0.003**	1.13 (1.03–1.23)	**0.006**	1.12 (1.03–1.22)	**0.008**
Feels dryness of eyes						
Never	*Ref*		*Ref*		*Ref*	
Sometimes	1.11 (0.99–1.24)	0.068	1.11 (0.98–1.24)	0.109	1.10 (0.98–1.23)	0.124
Always	1.05 (1.00–1.10)	**0.049**	1.04 (1.01–1.12)	**0.043**	1.05 (1.00–1.10)	**0.048**
Feels a foreign body in the eyes						
Never	*Ref*		*Ref*		*Ref*	
Sometimes	0.99 (0.94–1.04)	0.716	1.00 (0.95–1.05)	0.969	0.99 (0.95–1.05)	0.876
Always	1.28 (1.09–1.51)	**0.003**	1.26 (1.07–1.49)	**0.007**	1.26 (1.06–1.49)	**0.007**

CI: confidence interval; OR: odds ratio; Ref: reference

^a^Adjusted for age, gender, BMI, and living status

^b^Further adjusted for smoking and drinking habits

^c^Further additional adjustment for the number of remaining teeth and bite availability on right and left molars or with dentures

## Discussion

Previous studies have explored the potential links between PD and a range of eye diseases [2, 6, 9, 13, 14]. However, the evidence concerning the correlation between PD and dry eye remains limited. To our knowledge, this study represents the first large-scale population-based study into the impact of a history of dry eye disease or related symptoms on PD within the specific context of the Japanese population. Overall, the prevalence of PD in Japanese adults is considered high, with a significant gender difference in its history. However, there was no significant association found between DED and gender. The study revealed a significant association between the diagnosis or symptoms of dry eye and the history of PD among Japanese adults. Furthermore, significant associations were observed between PD and most variables, except for BMI and smoking habit.

Our study demonstrated that a range of DED diagnoses or related symptoms exhibited a significant association with PD, even after adjusting for potential confounding factors. A poorer periodontal status was more prevalent among individuals with a history of DED or related symptoms compared to those without such problems. While the exact causality remains uncertain in the existing literature, plausible explanations, including inflammatory processes, immunological factors, shared risk factors, medication use, and microbial translocation, have been suggested [2, 8, 9, 15–17]. In particular, it is worth noting that the inflammatory process, characterized by alterations in the cytokine network, may play a crucial role in linking these two conditions via a common inflammatory pathway [2, 16]. Previous studies have suggested that this pathogenic link might be mediated by shared immune responses and autoimmune diseases such as Sjögren’s syndrome, through the dysregulation of the immune system in both eye diseases and PD, potentially increasing susceptibility to inflammatory conditions [2, 18].

The pathogenesis of DED and PD can also be influenced by various common risk factors. Ageing, certain medications, and comorbidities such as diabetes, hypertension, as well as unhealthy behaviors like cigarette smoking and alcohol consumption, could be implicated in these associations [9, 19]. Dietary factors, such as vitamin deficiencies or imbalances in omega-3 fatty acids, may impact inflammation and contribute to the simultaneous occurrence of dry eye and PD [20, 21]. Moreover, individual factors such as genetics and lifestyle choices can further complicate the relationship between these two conditions. However, it is important to acknowledge that the complete mechanisms underpinning PD and DED are intricate and not yet fully understood. Therefore, further research is necessary to fully comprehend the underlying mechanisms and explore whether effectively managing one condition might have a positive impact on the other. Nevertheless, based on the findings of our study, we strongly advocate for early detection, targeted prevention, and effective management of both conditions. This entails the implementation of a multifaceted approach, encompassing good oral hygiene practices, overall health preservation, and mindful lifestyle choices.

The effects of demographic inequalities on oral health have been well documented. We observed a higher likelihood of PD development among older individuals and men in our study, which is consistent with findings in the literature supporting the widely recognized aging and gender differences in the development of PD [22, 23]. In the present study, it was observed that nearly half of the participants who lived alone had a history of PD, and living status showed a significant association with the likelihood of PD. Living status is recognized as a strong factor influencing individuals’ oral health [24]. Living alone is associated with a higher likelihood of developing depressive symptoms compared to living with family members [25]. Consequently, perceived psychological stressors have been identified as risk indicators for poor oral health, including periodontal diseases [26, 27].

Our study demonstrated that having fewer natural teeth and reduced chewing ability are associated with an increased risk of PD. This phenomenon can be attributed to a reduced self-cleaning mechanism and dietary changes [28]. Individuals with fewer teeth or reduced chewing ability may not effectively remove food remnants, and they may choose softer, processed foods that are higher in sugars and carbohydrates. This can foster the accumulation of plaque and bacteria, which are significant contributors to the development of PD. While the study identified a drinking habit as a significant associated factor for PD, smoking habit did not influence the likelihood of PD in the present study. This is perhaps because our survey only included general questions concerning these variables, omitting the duration and intensity of smoking. Consequently, respondents may have provided inaccurate or misleading responses.

This study has some limitations. First, while the sample is large enough, this study was carried out among Japanese adults residing in the Uonuma area of Niigata Prefecture, Japan. Therefore, this study could not be generalized to the entire Japanese population. Second, the potential presence of information bias may be considered. All measures utilized in this investigation relied on self-perceptions and self-reports. The determination of dry eye and periodontal disease was solely based on participants’ responses, without a current confirmation by a doctor or dentist. Third, this study utilizes data from the baseline of the Uonuma cohort study, which is inherently cross-sectional in design. Consequently, it is not possible to establish the direction of causality between the variables examined. Fourth, we did not perform missing data analysis in this study. Moreover, information pertaining to medication use and comorbidities could not be incorporated. Finally, it is important to acknowledge that in unforeseen circumstances, including delayed inter-researcher agreement, particularly during the COVID-19 pandemic, the completion of this study’s report was significantly delayed. Nevertheless, the study’s findings contribute valuable knowledge regarding the association between dry eye and periodontal disease, as the first large-scale epidemiological survey in Japan.

## Conclusion

Our study has revealed a significant correlation between dry eye and periodontal disease, suggesting that individuals with a history of dry eye disease or related symptoms were more likely to be associated with the development of subsequent periodontal disease. These findings underscore the importance of regular check-ups with both eye specialists and dentists, emphasizing the need for early detection and appropriate management of both conditions to ensure optimal health outcomes.

### Electronic supplementary material

Below is the link to the electronic supplementary material.

Supplementary Material 1

## Data Availability

The datasets generated and/or analyzed during the current study are not publicly available due to the restrictions by the Ethics Committee of Niigata University, Japan in order to protect the participants’ privacy but are available from the corresponding author on reasonable request.

## Abbreviations

PD: Periodontal Disease
DED: Dry Eye Disease
BMI: Body Mass Index
OR: Odds Ratio
95% CI: 95% Confidence Interval
