# Supplementary material for: Association between dry eye and periodontal disease in community-dwelling Japanese adults: data from the Uonuma cohort study
Source: BMC Oral Health. 2024 Jan 8;24:47. doi: 10.1186/s12903-023-03773-7 (PMC10775486; doi:10.1186/s12903-023-03773-7)
Supplement: Supplementary file 1 — Supplementary Material 1 [file 12903_2023_3773_MOESM1_ESM.docx]

**Supplementary Table 1.** Multivariable logistic regression analysis for periodontal disease in male participants

| Variables | Model 1^a^ | *p* value | Model 2^b^ | *p* value | Model 3^c^ | *p* value |
| --- | --- | --- | --- | --- | --- | --- |
|  | Adjusted OR (95%CI) |  | Adjusted OR (95%CI) |  | Adjusted OR (95%CI) |  |
| History of dry eye diagnosis  No  Yes | *Ref*  1.05 (0.92 – 1.20) | 0.452 | *Ref*  1.05 (0.92 – 1.20) | 0.448 | *Ref*  1.05 (0.92 – 1.21) | 0.442 |
| Feels dryness of eyes  Never  Sometimes  Always | *Ref*  1.12 (0.92 – 1.35)  1.06 (0.99 – 1.15) | 0.255  0.112 | *Ref*  1.12 (0.93 – 1.35)  1.06 (0.98 – 1.14) | 0.248  0.122 | *Ref*  1.12 (0.92 – 1.34)  1.06 (0.98 – 1.14) | 0.289  0.157 |
| Feels a foreign body in the eyes  Never  Sometimes  Always | *Ref*  0.97 (0.89 – 1.04)  1.31 (1.02 – 1.69) | 0.347  **0.038** | *Ref*  0.97 (0.89 – 1.04)  1.31 (1.02 – 1.69) | 0.357  **0.037** | *Ref*  0.97 (0.89 – 1.04)  1.31 (1.02 – 1.70) | 0.331  **0.036** |

CI: confidence interval; OR: odds ratio; Ref: reference.

^a^Adjusted for age, BMI, and living status.

^b^Further adjusted for smoking and drinking habits.

^c^Further additional adjustment for the number of remaining teeth and bite availability on right and left molars or with dentures.

**Supplementary Table 2.** Multivariable logistic regression analysis for periodontal disease in female participants

| Variables | Model 1^a^ | *p* value | Model 2^b^ | *p* value | Model 3^c^ | *p* value |
| --- | --- | --- | --- | --- | --- | --- |
|  | Adjusted OR (95%CI) |  | Adjusted OR (95%CI) |  | Adjusted OR (95%CI) |  |
| History of dry eye diagnosis  No  Yes | *Ref*  1.16 (1.03 – 1.32) | **0.018** | *Ref*  1.16 (1.02 – 1.32) | **0.020** | *Ref*  1.16 (1.02 – 1.31) | **0.023** |
| Feels dryness of eyes  Never  Sometimes  Always | *Ref*  0.96 (0.80 – 1.14)  1.03 (0.96 – 1.11) | 0.625  0.466 | *Ref*  0.96 (0.80 – 1.14)  1.03 (0.96 – 1.11) | 0.632  0.473 | *Ref*  0.96 (0.80 – 1.15)  1.03 (0.95 – 1.10) | 0.648  0.493 |
| Feels a foreign body in the eyes  Never  Sometimes  Always | *Ref*  0.96 (0.89 – 1.03)  1.17 (0.93 – 1.48) | 0.281  0.179 | *Ref*  0.96 (0.89 – 1.04)  1.18 (0.93 – 1.48) | 0.289  0.173 | *Ref*  0.96 (0.89 – 1.03)  1.17 (0.93 – 1.47) | 0.284  0.190 |

CI: confidence interval; OR: odds ratio; Ref: reference.

^a^Adjusted for age, BMI, and living status.

^b^Further adjusted for smoking and drinking habits.

^c^Further additional adjustment for the number of remaining teeth and bite availability on right and left molars or with dentures.
